# Probing the influence of PsbS on thylakoid lipid fluidity

**DOI:** 10.1007/s11120-026-01229-x

**Published:** 2026-07-17

**Authors:** Shiun-Jr Yang, Henry E. Lam, Katherine H. Richardson, Matthew P. Johnson, Gabriela S. Schlau-Cohen

**Affiliations:** 1 Department of Chemistry, Massachusetts Institute of Technology, 77 Massachusetts Ave, Cambridge, MA 02139 USA; 2 Plants, Photosynthesis and Soil, School of Biosciences, University of Sheffield, Firth Court, Western Bank, Sheffield, S10 2TN UK

**Keywords:** PsbS, Thylakoid membrane, Proteoliposome, Photoprotection, NPQ

## Abstract

Due to fluctuations in sunlight intensity, balancing light-harvesting efficiency and photoprotection is critical for plant fitness. To achieve this balance, plants have evolved a highly regulated mechanism, non-photochemical quenching (NPQ), which is activated under excess light to dissipate absorbed energy and deactivated under low light to preserve photosynthetic efficiency. A key component that allows NPQ to respond rapidly to changing light conditions is photosystem II subunit S (PsbS). Several hypotheses have been proposed to explain the working mechanism of PsbS, including the idea that it modulates thylakoid membrane macrostructure and protein mobility by altering membrane fluidity. To test this hypothesis, we employed a proteoliposome system containing only thylakoid lipids, PsbS, and the fluorescent probe laurdan, enabling direct detection of PsbS–lipid interactions under different pH conditions. Our results show that the presence of PsbS increases membrane fluidity, but the magnitude of this change in the overall bilayer is small compared with temperature-induced effects. These findings indicate that direct interactions between PsbS and thylakoid lipids are insufficient to account for NPQ regulation and suggest that interactions with other components in the thylakoid membranes are likely essential. This study provides insight into the functional mechanism of PsbS and establishes a framework for further investigation of PsbS-mediated regulation of NPQ and its role in optimizing photosynthetic performance under fluctuating light.

**Supplementary Information:**

The online version contains supplementary material available at 10.1007/s11120-026-01229-x.

## Introduction

Photosynthetic activity is highly dependent on sunlight intensity, which can undergo extreme fluctuations throughout the day. Under excess light conditions, photosynthesis can become limited by the downstream CO_2_ fixation reactions, leading to an accumulation of reducing pressure in the electron transfer chain (Li et al. 2009). The shortage of electron acceptors can lead to electron and energy transfer to oxygen at photosystem I and II (PSI and PSII, respectively), forming reactive oxygen species that have the capacity to damage the photosynthetic apparatus (Ruban et al. 2012; Degen and Johnson 2024). In PSII, the main risk of over-reduction of the electron transfer chain is the formation of triplet chlorophyll which can sensitize formation of singlet oxygen (Li et al. 2009). To prevent this, plants have evolved a highly regulated photoprotective mechanism to dissipate excess excitation energy in the PSII antenna light-harvesting complex II (LHCII), known as non-photochemical quenching (NPQ) (Ruban et al. 2012; Demmig-Adams et al. 2014; van Amerongen and Croce 2025). NPQ is comprised of several components that form and relax on different timescales. However, the major contributor is the rapidly-relaxing energy dependent quenching, qE (Ruban et al. 2012).

qE is activated via the build-up of the trans-thylakoid $\Delta\textrm{pH}$, which signals a saturation of the downstream CO_2_ fixation reactions, and is relaxed as $\Delta\textrm{pH}$ falls again in low light or darkness (Ruban et al. 2012; van Amerongen and Croce 2025). Accumulation of protons on the lumenal side of the thylakoid membrane leads to the protonation of the pH-sensing protein, PSII subunit S (PsbS) at two lumenal exposed glutamate residues (E122 and E226 in *Arabidopsis thaliana*) (Li et al. 2000, 2002, 2004; Marulanda Valencia and Pandit 2024). PsbS is an extremely hydrophobic transmembrane protein that does not bind pigments (Fan et al. 2015) and therefore cannot directly participate in the quenching mechanism. Rather, it has been proposed that protonation of E122 and E226 induces a dimer-to-monomer transition in PsbS, which then allows it to catalyze qE in LHCII (Bergantino et al. 2003; Pawlak et al. 2020).

At present, there is no consensus on how PsbS activates quenching pathways (Marulanda Valencia and Pandit 2024). On one hand, a recent molecular dynamics study suggested that the H3 motif of PsbS undergoes a conformational change from a turn/coil to a 3_10_ helix in response to $\Delta\textrm{pH}$ (Liguori et al. 2019). It was hypothesized that qE may be stably induced in LHCII through interaction with this 3_10_ helix on PsbS (Liguori et al. 2019; Krishnan-Schmieden et al. 2021). Indeed, recent in vivo data support this scheme in *Arabidopsis thaliana* (Chen et al. 2025). In this way, activated PsbS may induce conformational changes in LHCII, thereby opening quenching pathways within these complexes to dissipate excess excitation energy as heat (Wilk et al. 2013; Gerotto et al. 2015; Correa-Galvis et al. 2016; Daskalakis and Papadatos 2017; Sacharz et al. 2017; Liguori et al. 2019; Pawlak et al. 2020; Nicol and Croce 2021; Wilson et al. 2024).

A non-exclusive alternative for the mode of action of PsbS is that it influences the macrostructural reorganization of the thylakoid membrane that occurs during NPQ (Kiss et al. 2008; Betterle et al. 2009; Holzwarth et al. 2009; Kereïche et al. 2010; Johnson et al. 2011; Goral et al. 2012; Dong et al. 2015; Dall’osto et al. 2017; Daskalakis et al. 2019; Nishitani et al. 2026). For example, the presence of PsbS is required for the aggregation of LHCII trimers within the thylakoid membrane that was shown by freeze fracture electron microscopy to accompany qE formation, a feature that was linked to its ability to promote increased mobility of LHCII within the membrane (Johnson et al. 2011; Goral et al. 2012). Similarly, others have demonstrated that the absence of PsbS leads to an increased number of the PSII supercomplex semi-crystalline domains in the thylakoid membrane (Kiss et al. 2008; Kereïche et al. 2010; Nishitani et al. 2026). The opposite effects have also been observed for PsbS overexpressors, where LHCII showed increased mobility. Collectively, these observations suggest that PsbS promotes the reorganization in the thylakoid membrane, a process that is crucial for the activation of NPQ (Goral et al. 2012). This conclusion motivates the hypothesis that PsbS may regulate qE through its impact on membrane fluidity (Goral et al. 2012; Dong et al. 2015; Marulanda Valencia and Pandit 2024; Wilson et al. 2024).

To investigate the impact of PsbS on membrane fluidity, we employ a proteoliposome system containing PsbS. Proteoliposomes are widely used to study photosynthetic membrane proteins (Yang et al. 2006; Wilk et al. 2013; Liu et al. 2016; Pawlak et al. 2020; Tietz et al. 2020; Nicol and Croce 2021; Tutkus et al. 2021; Wilson et al. 2022; Manna et al. 2023) as they offer several advantages. First, they provide a simplified platform for isolating interactions between targeted proteins and lipids, in contrast to the native thylakoid membrane, where numerous additional components can complicate the analysis. Second, their composition can be controlled, allowing the stoichiometry of individual components to be tuned for quantitative studies. Third, they permit the incorporation of molecular probes into the membrane, enabling direct interrogation of protein–lipid interactions. In the experiments described below, we use a minimal system consisting solely of thylakoid lipids, PsbS, and the fluorescent dye, laurdan, to assess the impact of PsbS on membrane fluidity. Laurdan is widely used to probe lipid phase behavior in biological membranes (Parasassi et al. 1990, 1991; Parasassi and Gratton 1995; Szilágyi et al. 2008; Bagatolli 2013; Gunther et al. 2021), including liposomes, and its emission profile provides a quantitative measure of membrane fluidity.

## Materials and methods

### PsbS preparation

PsbS was overexpressed in Escherichia coli BL21 (DE3) and purified as described by Wilk et al. (2013). In brief, cell pellets were resuspended in 50 mM Tris pH 8.0, 730 mM sucrose, 1 mM EDTA, and treated with 10 mg lysozyme on ice for 30 min. Cells were lysed by microfluidizer (1100 psi) and centrifuged at $22,000 \times\textrm{g}$ for 20 min. The pellets were solubilised in 20 mM Tris pH 7.5, 1 mM *β*-mercaptoethanol, 0.5% Triton X-100 and inclusion bodies pelleted at $22,000 \times\textrm{g}$ for 20 min. Inclusion bodies were solubilised in 50 mM HEPES pH 8, 2% (wt/vol) lithium dodecyl sulfate (LDS), and 8 M urea, then centrifuged for 5 min at $20,000\times\textrm{g}$. The supernatant was loaded onto His-select Nickel Affinity Gel (Sigma) and washed with 50 mM HEPES pH 8, 0.1% LDS. PsbS was eluted by lowering the pH to 5.3 and then mixed with an equal volume of 0.1 M HEPES pH 7.5, 4% LDS, and 0.73 M sucrose, and heated to $100^\circ\textrm{C}$ for 1 min. Detergent was exchanged by addition of 1% n-octyl-*β*-D-glucopyranoside (OG; Glycon Biochemicals GmbH) and LDS was precipitated with 200 mM KCl. Samples were centrifuged for 10 min at $20,000\times\textrm{g}$. The eluate was further concentrated by ultrafiltration [Vivaspin (Sartorius); molecular weight cutoff (MWCO) = 10 kDa] and repeatedly washed with 50 mM HEPES pH 7.5, 1% OG. Samples were flash-frozen in liquid nitrogen and stored at $-80^\circ\textrm{C}$. A circular dichroism (CD) spectrum of the purified PsbS is shown in the Supplementary Information. The CD spectrum matches those reported previously (Aspinall-O’Dea et al. 2002; Wilk et al. 2013), indicating the protein is folded correctly.

### Proteoliposome preparation

Thylakoid lipids, monogalactosyldiacylglycerol (MGDG), digalactosyldiacylglycerol (DGDG), sulfoquinovosyldiacylglycerol (SQDG), and L-*α*-phosphatidylglycerol (Soy PG) were purchased from Avanti Polar Lipids, Inc. Lipids were suspended in 7:3 (v/v) chloroform:methanol mixture and combined at the following molar ratio: 50% MGDG, 30% DGDG, 10% SQDG, 10% Soy PG. Laurdan (Thermo Fisher Scientific) was dissolved in chloroform and added to the lipid mixture with a laurdan:lipid molar ratio of 1:100. The lipid-laurdan mixture was aliquoted into 0.5 mg portions, purged with $\textrm{N}_2$ gas, and transferred to a vacuum desiccator for 2 hrs. The vials were then purged with $\textrm{N}_2$ gas again, sealed, and stored at $-70^\circ\textrm{C}$ until use.

To form proteoliposomes, the lipid-laurdan mixture was resuspended in working buffer (20 mM HEPES, 40 mM NaCl, pH 7.5) for 30 mins. The suspension underwent three freeze-thaw cycles, and was subsequently extruded 19 times through a 50 nm polycarbonate membrane using a Mini-Extruder (Avanti Polar Lipids). Formation of empty liposomes was confirmed by dynamic light scattering (DLS). Liposomes were then destabilized by the addition of 0.03% (w/v) *β*-DDM, followed by incubation on ice for 30 mins with occasional gentle mixing. PsbS was added at protein-to-lipid molar ratios (P/L) ranging from 1:500 to 1:100. The mixture was incubated on ice for 1 hr with occasional gentle mixing and subsequently subjected to dialysis for 42 hr against working buffer (20 mM HEPES, 40 mM NaCl, pH 7.5) using Slide-A-Lyzer MINI Dialysis Devices (Thermo Fisher Scientific). Unincorporated protein was removed by centrifugation at 15,000 rpm ($21,382\times\textrm{g}$) for 15 min (Nicol and Croce 2021). The supernatant containing proteoliposomes was collected and passed through a $0.22\mu\textrm{m}$ polycarbonate filter (Millex-GV, Merck Millipore Ltd.). Formation of PsbS liposome was confirmed by DLS prior to laurdan emission measurements.

The pH of both empty liposome and PsbS liposome was lowered by buffer exchange into a low-pH buffer (20 mM HEPES, 40 mM NaCl, pH 5.5). Samples were diluted with the low-pH buffer and reconcentrated by centrifugation at 8,000 rpm for 5 mins using a 30 kDa MWCO centrifugal filter (Amicon Ultra 0.5, Merck Millipore Ltd.). This dilution-concentration cycle was repeated for three times, after which the final concentrations of all samples were adjusted to match those prior to buffer exchange. Samples were incubated for 3 hrs, after which liposome formation was confirmed by DLS and the laurdan emission spectra were collected.

### Characterization and quantification

The size distributions of empty and PsbS liposomes at pH 7.5 and pH 5.5 were determined by DLS using a Prometheus Panta instrument (NanoTemper). The mean particle diameters were consistently in the range of 50–70 nm for all samples, and the polydispersity index (PDI) remained below 0.15, indicating a narrow size distribution. Representative size distributions of empty and PsbS liposomes at pH 7.5 and pH 5.5 are shown in the Supplementary Information (Figure S2). Laurdan emission spectra were collected using a commercial fluorescence spectrophotometer (Carry Eclipse, Agilent). The excitation wavelength was set to 355 nm. For temperature-dependent measurements, a temperature control module (CoolSpeK USP-203-B, UNISOKU) was integrated into the spectrophotometer.

Quantification of PsbS insertion efficiency was performed by SDS-PAGE analysis followed by Coomassie Brilliant Blue staining. For each proteoliposome preparation, the stock PsbS solution used for reconstitution was also loaded on the gel in four separate lanes at volumes $2\mu\textrm{L},$ $1\mu\textrm{L}$, $0.5\mu\textrm{L}$, and $0.2\mu\textrm{L}$. These standards were used to construct a calibration curve, from which the relative amount of PsbS incorporated into the liposomes was interpolated. Stained gels were imaged using a ChemiDoc MP Imaging System (Bio-Rad). More details can be found in the Supplementary Information.

## Results

**Fig. 1:**
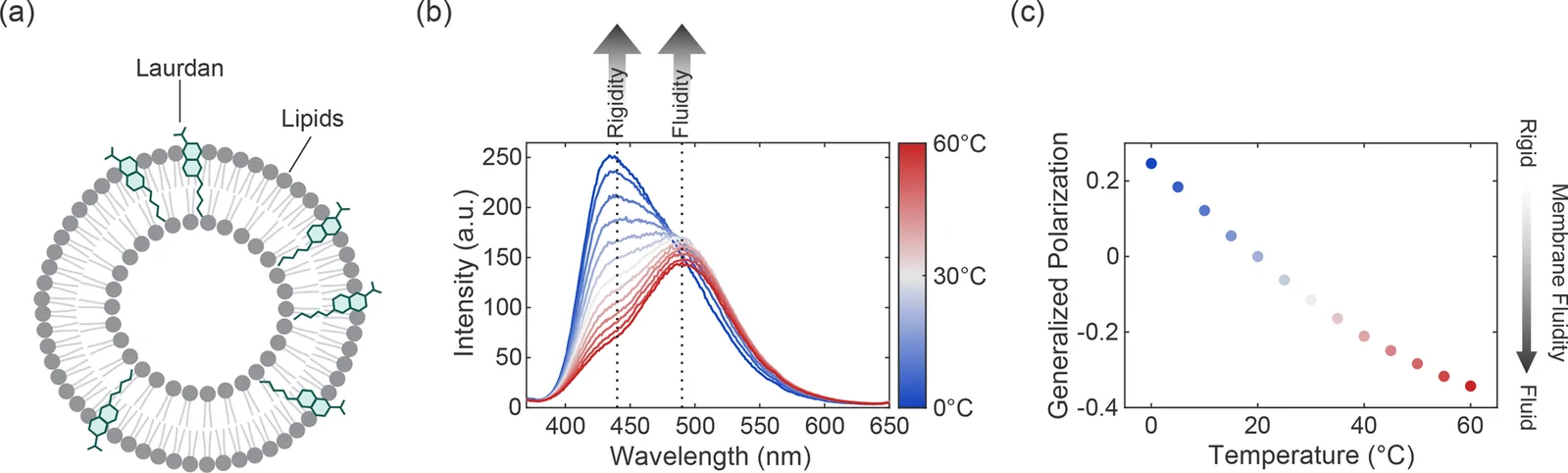
(**a**) A schematic of an empty liposome with laurdan molecules incorporated in the lipid bilayer. (**b**) Temperature-dependent laurdan emission when incorporated in the empty liposome composed of thylakoid lipids. The peaks at 440 nm and 490 nm (dotted lines) correspond to the gel phase (more rigid) and liquid crystalline phase (more fluid), respectively. (**c**) Generalized polarization (GP) extracted from the laurdan emission spectra in (**b**) shown in the corresponding colors

A typical emission spectrum of laurdan in membranes consists of two components that correspond to environments of different polarity, which are strongly correlated with lipid phase. Specifically, emission in gel-phase membranes, which are more rigid and allow less reorganization, is blue-shifted, whereas emission in the liquid-crystalline-phase membranes, which are more fluid and allow more reorganization, is red-shifted. Figure 1b shows the temperature-dependent emission spectra of laurdan incorporated in liposomes composed of thylakoid lipids over a temperature range from $0^\circ\textrm{C}$ to $60^\circ\textrm{C}$. The spectra exhibit two components centered approximately at 440 nm and 490 nm, corresponding to the gel and liquid-crystalline phase, respectively. The coexistence of these lipid phases can be quantitatively described by the generalized polarization (GP), defined as (Parasassi et al. 1990, 1991; Parasassi and Gratton 1995)

1 $$\mathrm{GP} = \frac{I_{440}-I_{490}}{I_{440}+I_{490}}$$

where $I_{440}$ and $I_{490}$ are the emission intensities at 440 nm and 490 nm. Within this framework, a higher GP value (higher $I_{440}$) indicates a more rigid membrane environment, whereas a lower GP value (higher $I_{490}$) indicates a more fluid membrane.

Figure 1c shows the GP values extracted from the temperature-dependent spectra of laurdan in the thylakoid lipid liposomes. At low temperatures, the model membrane system composed of thylakoid lipids exhibits higher GP values, consistent with a more rigid membrane characterized by stronger emission at 440 nm. As temperature increases, the membrane becomes progressively more fluid, resulting in lower GP values. Over the temperature range from $0^{\circ}$ to $60^{\circ}C$, the GP decreases from 0.25 to −0.34. These values provide a quantitative reference for the extent of membrane fluidity changes that thylakoid lipid membranes can undergo under physiologically relevant temperature fluctuations.

**Fig. 2:**
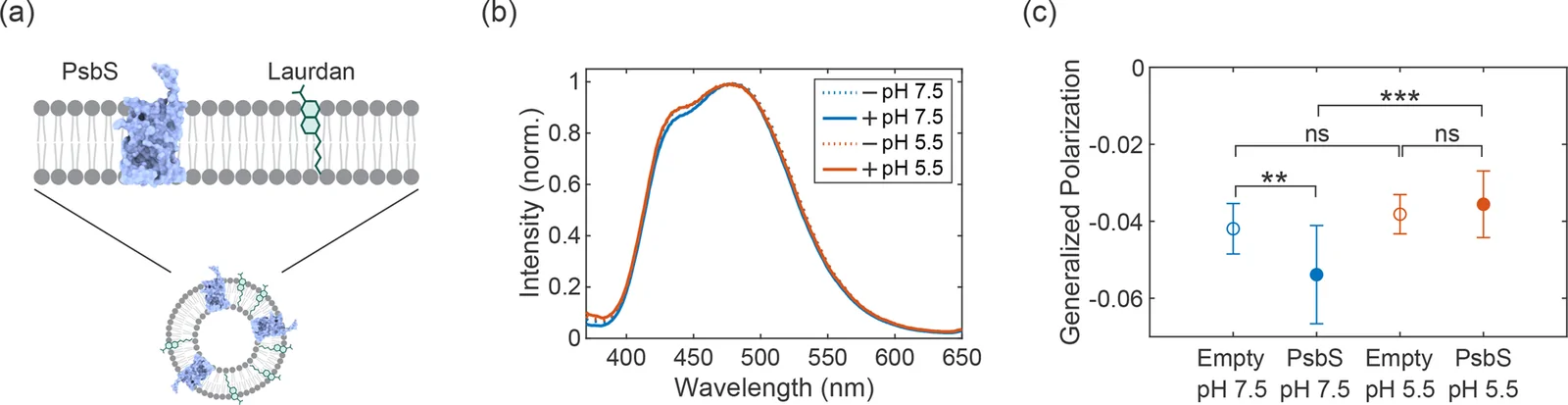
(**a**) A schematic of PsbS proteoliposomes with laurdan molecules co-incorporated. (**b**) Averaged laurdan emission in empty (-, dotted lines) and PsbS (+, solid lines) liposome at pH 7.5 (blue) and pH 5.5 (red). (**c**) GP values extracted from (**b**) With error bars showing the standard deviations of 15 replicates. Asterisks indicate significant differences between the GP values, assessed by Student’s *t*-test. (ns, *p > 0.05*; **, *p < 0.01*; ***, *p < 0.001*; *n=15*)

To assess the impact of PsbS and pH on membrane fluidity, we compare laurdan emission spectra and corresponding GP values for liposomes with and without PsbS at pH 7.5 and pH 5.5. Figure 2b shows the normalized laurdan emission spectra of the different liposome samples measured at room temperature. Among these, only the PsbS liposomes at pH 7.5 exhibits a visibly distinct spectrum, characterized by a reduced emission intensity at 440 nm. Figure 2c summarizes the averaged GP values and standard deviations obtained from 15 independent replicates for each condition. The GP values for empty liposomes at both neutral and low pH, as well as for PsbS liposomes at low pH, fall within a narrow range between −0.035 and −0.042. In contrast, the GP value for PsbS liposomes at pH 7.5 is −0.053. Statistical analysis using Student’s *t*-tests indicates that the incorporation of PsbS into liposomes results in a statistically significant decrease in GP at neutral pH. Lowering the pH in the presence of PsbS increases the GP, restoring it to a level comparable to that of empty liposomes at low pH. In contrast, no statistically significant change in GP is observed upon lowering the pH for empty liposomes.

Although the averaged laurdan emission spectra and GP values show that the presence of PsbS increases membrane fluidity, it is important to interpret these results at a molecular level in order to assess whether the observed effect is relevant for qE regulation. In particular, the spatial relationship between laurdan and PsbS must be considered to quantify the extent of membrane fluidity changes induced by PsbS. To estimate the distance between laurdan and PsbS, we experimentally determine the number of PsbS proteins per liposome and combine this information with numerical simulation. First, the PsbS incorporation efficiency is estimated using SDS-PAGE analysis followed by Coomassie Brilliant Blue staining. We find that the protein incorporation efficiency varies between 35% to 100% across different preparations and shows no clear dependence on the initial protein-to-lipid mixing ratio (P/L) within the range of 1:100 to 1:500. These observations are consistent with previous reports on LHCII liposomes (Tutkus et al. 2018).

**Fig. 3:**
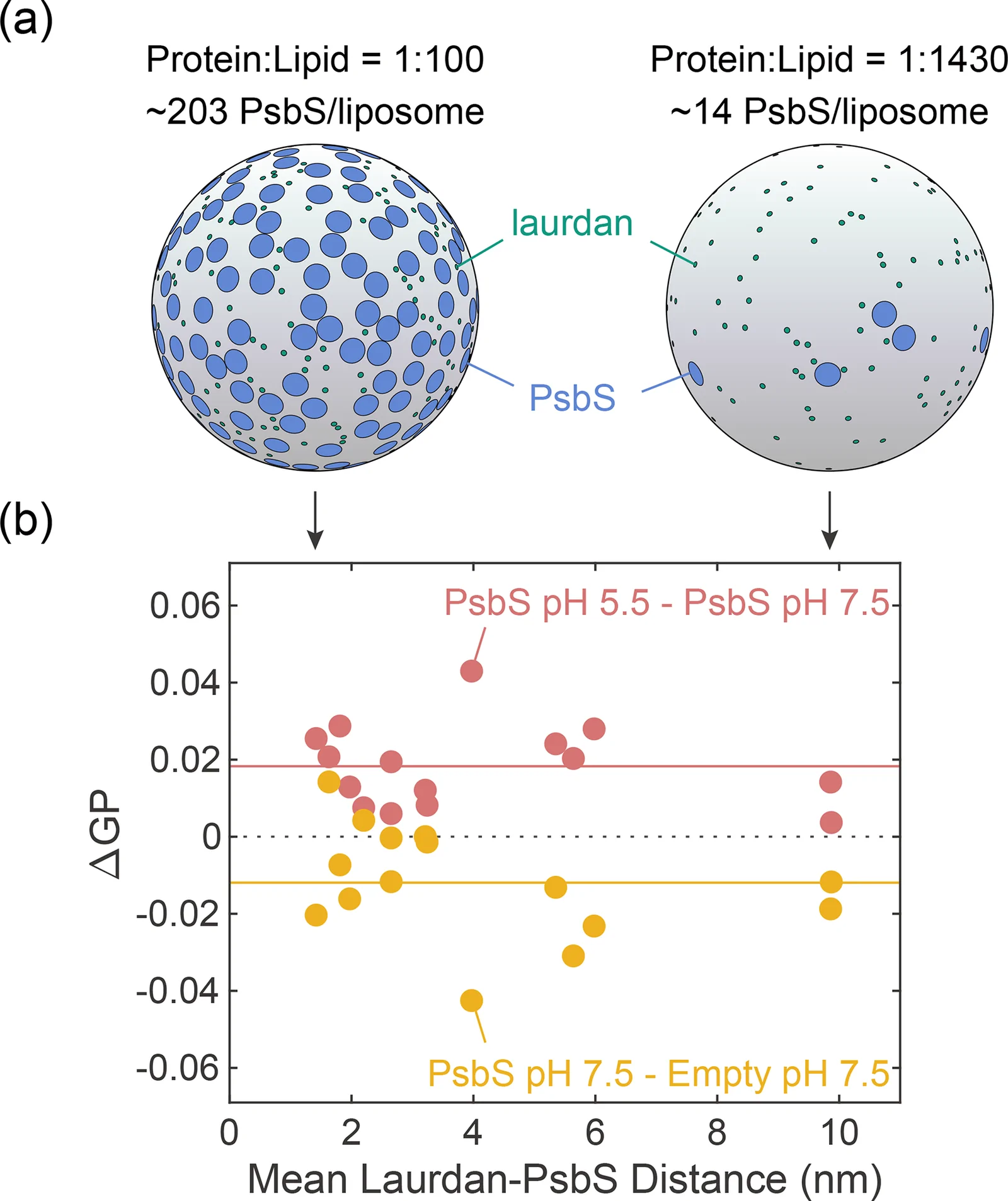
(**a**) Simulated liposomes with randomly distributed PsbS (blue) and laurdan molecules (green) at P/L of 1:100 (left, *∼ 203* PsbS per liposome) and 1:1430 (right, *∼ 14* PsbS per liposome), which correspond to the shortest and longest distances in (**b**). The laurdan:lipid ratio is 1:100. (**b**) Differences of the GP between PsbS and empty liposome at pH 7.5 (yellow) and PsbS liposome at pH 7.5 and pH 5.5 (red) at different distances between laurdan and nearest PsbS. Solid lines show the mean GP difference across the 15 replicates and the dotted line shows zero GP difference

Based on the incorporation efficiency, we estimate the effective P/L and, consequently, the number of PsbS proteins per liposome for each replicate. These values are used as input parameters for simulations of PsbS liposomes. In the simulations, the corresponding number of PsbS proteins and laurdan molecules are randomly distributed on the surface of a sphere with a diameter matched to that of PsbS liposomes. Figure 3a shows two representative simulated PsbS-laurdan liposomes corresponding to the two extremes of effective P/L (1:100 and 1:1430). From these simulations, the surface distance between each laurdan molecule and its nearest PsbS protein is calculated. By repeating the simulation multiple times and evaluating the nearest-neighbor distances for all laurdan molecules, we construct distributions of laurdan-PsbS distances for each effective P/L.

Figure 3b shows the GP changes as a function of the mean laurdan-PsbS distance. Owing to variations in both the initial mixing P/L and the PsbS incorporation efficiencies across preparations, the effective P/L spans a range from 1:100 to 1:1430, corresponding to *∼ 203* and *∼ 14* PsbS per liposome and mean laurdan-PsbS distances of 1.4 nm and 9.9 nm, respectively. Within this range, neither the GP decrease associated with PsbS insertion (yellow) nor the GP increase induced by lowering pH in the presence of PsbS (red) exhibits a clear dependence on the mean laurdan-PsbS distance, despite the nonzero average differences indicated by the solid lines. This observation suggests that the impact of PsbS on membrane fluidity is either saturated within this distance range or not additive.

It is also possible that PsbS and laurdan are not randomly distributed within the lipid bilayer. For example, PsbS and laurdan may preferentially associate with each other, leading to shorter actual laurdan-PsbS distances than those predicted by the simulations. In this case, different effective P/L would correspond to similar mean laurdan-PsbS distances, potentially accounting for the lack of observed distance dependence. Under such conditions, the effect of PsbS on membrane fluidity would be expected to be saturated even at relatively low effective P/L ratios. However, this model requires an extremely large interaction energy due to the entropic driving force for laurdan to diffuse through the lipid bilayer, particularly at low effective P/L ratios. All components are similarly very hydrophobic within the transmembrane region, making such a preferential interaction energy challenging to rationalize.

## Discussion

Previous experiments using wild-type *Arabidopsis thaliana* and PsbS knockout and overexpressing mutants (*npq4* and L17, respectively) have demonstrated that the macrostructure and protein mobility of the thylakoid membrane are correlated with PsbS abundance (Goral et al. 2012). In particular, the absence of PsbS leads to increased formation of PSII arrays and reduced protein mobility in dark-adapted chloroplasts, whereas overexpression of PsbS produces the opposite effect. These observations showed that PsbS plays an essential role for membrane reorganization, and it was proposed that PsbS acts as a “lubricant” that maintains the fluidity of the densely packed thylakoid membrane. Consistent with this hypothesis, our model system, which is comprised of only thylakoid lipids, PsbS, and laurdan, shows that the presence of PsbS leads to lower GP values, indicating a more fluid membrane environment. While this finding qualitatively agrees with the observations of Goral et al. (2012), the magnitude of the effect is small: the averaged GP decreases by only 0.018 upon PsbS insertion. Due to spatial heterogeneity, it is possible that local fluidity change near PsbS is greater than what this ensemble GP change reflects. In this case, however, it is expected that increasing the number of PsbS per liposome, which shortens the mean laurdan-PsbS distance and increases the number of local effects, should result in a larger overall GP change. The lack of dependence on the mean laurdan-PsbS distance shown in Fig. 3b suggests that local effect is not significantly greater than what the ensemble GP reports, potentially due to the preferential association between PsbS and laurdan mentioned above. The change in membrane fluidity due to the presence of PsbS is also exclusive to pH 7.5, which may reflect a difference in PsbS dimerization state. This change is minimal when compared to the effect of temperature, which has a more pronounced influence on membrane fluidity. Indeed, a GP decrease of comparable magnitude can be induced by a temperature increase of approximately $1.2^{\circ}\textrm{C}$, as interpolated from Fig. 1c. This comparison suggests that the direct interactions between PsbS and thylakoid lipids alone are unlikely to be sufficient to account for qE regulation. If they were, typical environmental temperature fluctuations, which often exceed $1.2^{\circ}$C, would be expected to induce comparable changes in thylakoid membrane macrostructure and protein mobility, thereby interfering with the light-dependent control of qE.

Although our results indicate that the membrane fluidity change directly induced by PsbS is insufficient for qE regulation, PsbS may still modulate membrane properties indirectly through interactions with other components in the thylakoid membrane. In particular, recent molecular dynamics (MD) simulations have shown that interactions between LHCII and lipids are altered in the presence of PsbS (Daskalakis et al. 2019). Specifically, in the absence of PsbS, lumen acidification promotes binding of LHCII to DGDG, which can lead to immobilization of LHCII within the membrane. In contrast, when PsbS is present, lumen acidification favors the formation of PsbS-LHCII complexes, disrupting the accumulation of DGDG around LHCII and preserving its mobility. This picture is further supported by experimental observations showing that DGDG fluxes correlate with the presence of PsbS and, consequently, with the qE response (Wilson et al. 2024). Together, these studies indicate that lipid composition in the vicinity of LHCII plays a critical role in controlling both LHCII mobility and thylakoid membrane fluidity, and is therefore central to qE regulation. It is important to note that the activation of qE is usually accompanied by the xanthophyll cycle, in which violaxanthin is converted to zeaxanthin upon lumen acidification. While the presence of zeaxanthin has been shown to modulate membrane fluidity (Szilágyi et al. 2008), PsbS is still an essential component for light-induced membrane reorganization and qE activation (Goral et al. 2012; Nishitani et al. 2026).

It should be noted that laurdan reports primarily on lipid packing order and headgroup hydration rather than bilayer thickness directly. The small GP changes observed here therefore do not exclude the possibility that PsbS exerts a more substantial effect on bilayer thickness that is not captured by the laurdan assay, particularly since thickness changes have been reported to accompany qE in vivo (Johnson et al. 2011; Wilson et al. 2024). The rigidity and thickness of the membrane are not necessarily completely coupled in the presence of transmembrane proteins whose hydrophobic span can impose local thickness constraints independently of bulk fluidity. It has been shown, however, that rigidity and thickness of a membrane are positively correlated in simple systems (Bermúdez et al. 2004), although the relationship is also highly influenced by additional factors (Evans and Needham 1987), including lipid composition (Rawicz et al. 2000) and interactions with proteins (Kondrashov and Akimov 2023).

In principle, proteoliposomes containing LHCII, PsbS and laurdan would provide an ideal platform for directly investigating the interplay between protein interactions and membrane fluidity under conditions that mimic photoprotection. However, laurdan emission significantly overlaps with the Soret band of LHCII, raising the possibility of energy transfer from laurdan to LHCII. Previous studies have demonstrated that excitation energy can be transferred from chromophores embedded in model membrane systems to nearby light-harvesting complexes over distances of only a few nanometers (Hancock et al. 2019, 2021). Given that the mean laurdan-protein distance in our proteoliposomes is likely within this nanometer range, it is very likely that excitation energy transfer from laurdan to LHCII would occur. Such energy transfer would alter the laurdan emission spectrum, causing the extracted GP values to no longer faithfully report membrane fluidity. In fact, preliminary tests showed spectral signatures of energy transfer pathways from laurdan to LHCII, which could not be robustly separated from spectral signatures reporting on the membrane properties. These observations suggest that an ideal membrane fluidity probe for studying systems containing LHCII would emit in the near-infrared region, where LHCII absorption is minimal, as LHCII absorbs across nearly the entire visible spectrum.

## Conclusion

PsbS plays a crucial role in the regulation of qE and is directly linked to plant growth, yet its molecular mechanism remains unresolved. In this work, we test the hypothesis that PsbS regulates qE through direct modulation of thylakoid membrane fluidity using a minimal proteoliposome system containing thylakoid lipids, PsbS, and the fluorescent probe laurdan. We find that although incorporation of PsbS leads to a statistically significant increase in membrane fluidity, the magnitude of this effect is quantitatively small and physiologically negligible when compared with temperature-induced changes. This result establishes an upper bound on the extent to which direct PsbS–lipid interactions can contribute to qE regulation, indicating that such interactions alone are insufficient to account for the observed light-dependent control of photoprotection. Instead, our findings support a model in which PsbS modulates membrane organization indirectly, likely through interactions with other thylakoid components such as LHCII. With an improved membrane fluidity probe, the proteoliposome platform presented here provides a versatile framework for dissecting protein–protein and protein–lipid interactions that underlie qE and thylakoid membrane reorganization. Ultimately, understanding these molecular mechanisms is critical for elucidating how qE contributes to photoprotection and, more broadly, to plant fitness under fluctuating light environments.

## Electronic supplementary material

Below is the link to the electronic supplementary material.

Supplementary Material 1

## Data Availability

All data supporting the findings of this study are available at 10.5281/zenodo.20172066.

## Code availability

The code used for liposome simulations is available at 10.5281/zenodo.20172066.
