# Supplementary material for: Probing the influence of PsbS on thylakoid lipid fluidity
Source: Photosynth Res. 2026 Jul 17;164(4):40. doi: 10.1007/s11120-026-01229-x (PMC13379455; doi:10.1007/s11120-026-01229-x)
Supplement: Supplementary file 1 — Supplementary Material 1 [file 11120_2026_1229_MOESM1_ESM.pdf]

Supplementary Information for Probing the influence of PsbS on thylakoid lipid fluidity

Shiun-Jr Yang[1], Henry E. Lam[1], Katherine H. Richardson[2], Matthew P. Johnson[2*], Gabriela S. Schlau-Cohen[1*]

[1*]Department of Chemistry, Massachusetts Institute of Technology, 77 Massachusetts Ave, Cambridge, 02139, MA, USA.
[2*]Plants, Photosynthesis and Soil, School of Biosciences, University of Sheffield, Firth Court, Western Bank, Sheffield, S10 2TN, State, U.K..

*Corresponding author(s). E-mail(s): matt.johnson@sheffield.ac.uk; gssc@mit.edu;
Contributing authors: yangsj@mit.edu; lamhenry@mit.edu; katherine.richardson@sheffield.ac.uk;

Sample Characterization

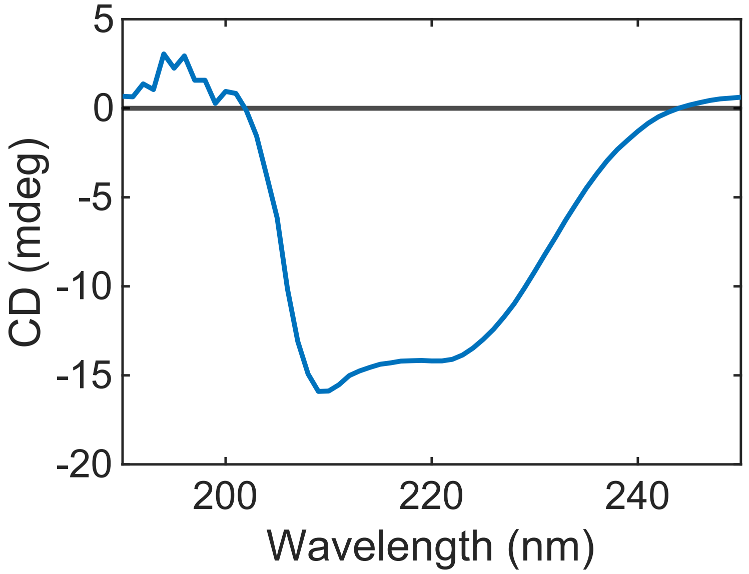

Fig. S1 Circular dichroism (CD) spectrum of PsbS. The spectrum was measured on a Jasco J-810 spectrophotometer at a protein concentration of 0.1% (50 mM HEPES pH 7.5, 1% OG), in a cell with 50 mm pathlength at 20°C. The assay was performed at a wavelength range of 190–250 nm with values recorded at 1-nm intervals. mdeg: millidegrees.

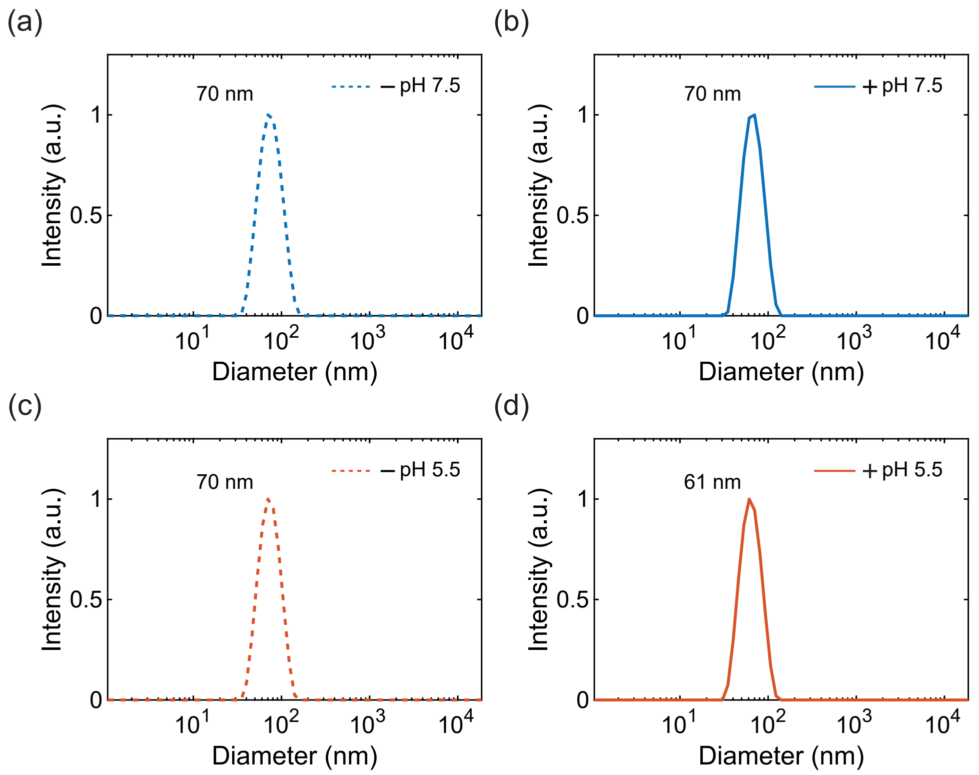

Fig. S2 Representative size distributions of (a) empty liposome at pH 7.5, (b) PsbS liposome at pH 7.5, (c) empty liposome at pH 5.5, and (d) PsbS liposome at pH 5.5, obtained from dynamic light scattering (DLS) measurements.

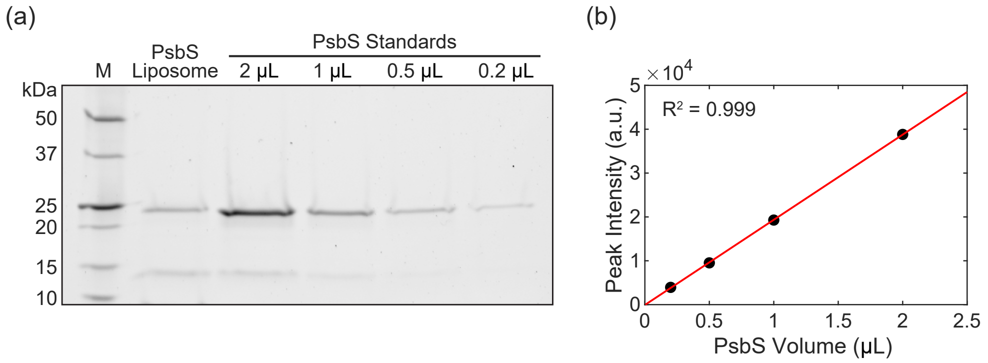

Fig. S3 (a) A representative SDS-PAGE analysis of PsbS liposomes and PsbS standards followed by Coomassie Brilliant Blue staining. The M lane shows marker proteins with the indicated molecular masses. (b) The peak intensities of the PsbS standards at around 22 kDa extracted from (a). The fitted line is shown in red.

PsbS incorporation efficiency was estimated using SDS-PAGE analysis followed by Coomassie Brilliant Blue staining. Figure S3a shows a representative gel containing marker proteins, PsbS liposomes, and PsbS standards. The PsbS standards were prepared from the same stock sample used for proteoliposome formation. Standards containing 2 μL, 1 μL, 0.5 μL, and 0.2 μL of PsbS stock were diluted to match the final volume of the PsbS liposome sample used for the SDS-PAGE analysis. Figure S3b shows the calibration curve constructed from the band intensities of the gel in Figure S3a. The data points were fitted with a linear function, which was then used to estimate the PsbS incorporation efficiency.

PsbS-laurdan Liposome Simulation

Numerical simulations were performed to estimate the mean laurdan-PsbS surface distance at a given effective protein-to-lipid ratio (P/L), assuming that laurdan and PsbS are randomly distributed within the lipid bilayer without overlap. In the simulations, laurdan-PsbS distances were calculated based on the liposome diameter, the number of proteins per liposome, and the number of laurdan molecules per liposome.

In principle, the liposome diameter can be obtained experimentally from DLS measurements, as shown in Figure S2. However, DLS determines the hydrodynamic diameter rather than the core diameter of the liposomes. Therefore, the liposome diameter was set to 50 nm, corresponding to the pore size of the polycarbonate membrane used during extrusion (see Materials and Methods). This approximation is consistent with DLS measurements showing mean hydrodynamic diameters between 50 and 70 nm.

Using the liposome diameter, the number of lipids per liposome was estimated, which is required to determine the number of proteins and laurdan molecules per liposome. The number of lipids per liposome was estimated using the following expression:

$$N_{\text{lipid}} = \frac{4\pi(\frac{d}{2})^2 + 4\pi(\frac{d}{2} - t)^2}{a} \tag{S1}$$

In Equation S1, N_{lipid} is the number of lipids per liposome, d is the core diameter of the liposomes, t is the thickness of the lipid bilayer, and a is the area per lipid molecule. In the simulations, d was set to 50 nm, t was set to 4 nm (Kirchhoff et al. 2011; Wilson et al. 2024), and a was set to 0.66 nm^2 (van Eerden et al. 2015; Tietz et al. 2020). These parameters yield $N_{\text{lipid}} \approx 2.0 \times 10^4$ and subsequently allow estimation of the number of proteins and laurdan molecules per liposome for a given effective P/L and laurdan-to-lipid ratio, the latter being fixed at 1:100 through experimental control.

The two extreme cases with the highest and lowest effective P/L are shown in Figure 3a in the main text. The effective P/L was calculated from the mixing ratio (experimentally controlled) and the insertion efficiency (experimentally determined). Here, we use an example with a mixing P/L of 1:500 and an insertion efficiency of 70 % to demonstrate how the mean laurdan-PsbS distance is obtained in the simulations. First, the effective P/L is calculated as $1 \times 70\% : 500 \approx 1 : 714$ (Figure S4a). Based on this ratio and the N_{lipid} obtained from Equation S1, the number of PsbS proteins per liposome is estimated to be ∼28 and the number of laurdan molecules per liposome is estimated to be ∼203. The corresponding numbers of PsbS and laurdan molecules are then randomly distributed on a spherical surface with a diameter of 50 nm. The molecular areas of PsbS and laurdan were set to 12.4 nm^2 and 0.6 nm^2, respectively (Fan et al. 2015). We note that the calculated laurdan-PsbS distances are not sensitive to moderate variations in the assumed molecular areas, because the liposomes are not densely packed, and the nearest-neighbor distances are governed primarily by surface number density.

At this stage, a simulated PsbS-laurdan liposome is generated. To obtain the laurdan-PsbS distance distribution, 10,000 liposomes with randomly distributed PsbS and laurdan molecules were simulated. For each laurdan molecules in all simulated liposomes, the nearest PsbS protein was identified, and the distance between the

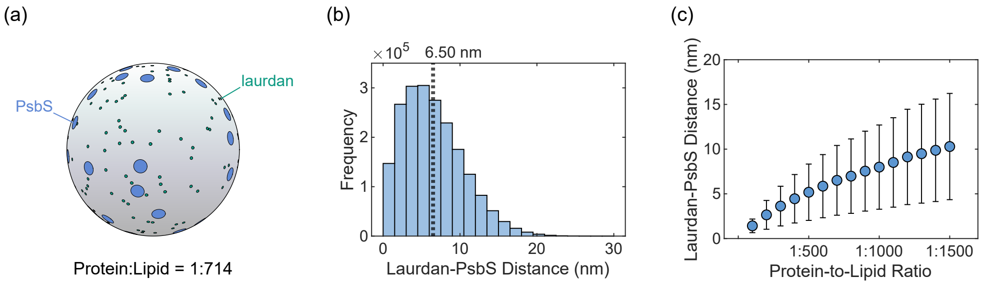

Fig. S4 (a) Simulated liposomes with randomly distributed PsbS (blue) and laurdan molecules (green) at a P/L of 1:714. (b) Laurdan-PsbS distance distribution at a P/L of 1:714 obtained from 10,000 simulated liposome. The dotted line indicates the mean distance (6.5 nm). (c) Mean laurdan-PsbS distance at different P/L ratios. Error bars correspond to the standard deviation of the laurdan-PsbS distance distribution.

laurdan molecule and the PsbS protein was calculated. Figure S4b shows the laurdan-PsbS distance distribution obtained from the 10,000 simulated liposomes with an effective P/L of 1:714 (corresponding to a mixing P/L of 1:500 and an insertion efficiency of 70 %). A mean laurdan-PsbS distance of 6.5 nm was extracted from the distribution and used for Figure 3b in the main text. Figure S4c shows the mean laurdan-PsbS distances at effective P/L ratios between 1:100 and 1:1500, along with the corresponding standard deviations extracted from the distributions. All PsbS-laurdan liposomes reported in the main text have effective P/L ratios within this range. The corresponding mean laurdan-PsbS distances and the standard deviations at P/L ratios of 1:100 and 1:1500 are 1.42 ± 0.76 nm and 10.29 ± 5.94 nm, respectively.

References

Fan M, Li M, Liu Z, et al (2015) Crystal structures of the psbs protein essential for photoprotection in plants. Nature structural & molecular biology 22(9):729–735. https://doi.org/10.1038/nsmb.3068

Kirchhoff H, Hall C, Wood M, et al (2011) Dynamic control of protein diffusion within the granal thylakoid lumen. Proceedings of the National Academy of Sciences 108(50):20248–20253. https://doi.org/10.1073/pnas.1104141109

Tietz S, Leuenberger M, Höhner R, et al (2020) A proteoliposome-based system reveals how lipids control photosynthetic light harvesting. Journal of Biological Chemistry 295(7):1857–1866. https://doi.org/10.1074/jbc.RA119.011707

van Eerden FJ, de Jong DH, de Vries AH, et al (2015) Characterization of thylakoid lipid membranes from cyanobacteria and higher plants by molecular dynamics simulations. Biochimica et Biophysica Acta (BBA) - Biomembranes 1848(6):1319–1330. https://doi.org/10.1016/j.bbamem.2015.02.025

Wilson S, Clarke CD, Carbajal MA, et al (2024) Hydrophobic mismatch in the thylakoid membrane regulates photosynthetic light harvesting. Journal of the American Chemical Society 146(21):14905–14914. https://doi.org/10.1021/jacs.4c05220
